# Should Lifestyles Be a Criterion for Healthcare Rationing? Evidence from a Portuguese Survey

**Published:** 2017-11-18

**Authors:** Ana Pinto Borges, Micaela Pinho

**Affiliations:** ^1^ Department of Economic and Management, School of Management, University of ISAG European Business School, Porto, Portugal & Department of Economic, Faculty of Economics and Business Sciences, University of Lusíada - North; ^2^ Economics, Management and Informatic Department; University Portucalense, OPorto, Portugal & Economics, Management, Industrial Engineering and Tourism Department; University of Aveiro, Aveiro, Portugal

**Keywords:** Rationing healthcare, Risk behaviours, Health responsibility, Priority setting, Portugal

## Abstract

**Background:** We evaluated whether different personal responsibilities should influence the allocation healthcare resources and whether attitudes toward the penalization of risk behaviours vary among individual’s sociodemographic characteristics and health related habits.

**Study design:** A cross-sectional study.

**Methods:** We developed an online survey and made it available on various social networks for six months, during 2015. The sample covered the population aged 18 yr and older living in Portugal and we got 296 valid answers. Respondents faced four lifestyle choices: smoking, consumption of alcoholic beverages, unhealthy diet and illegal drug use, and should decide whether each one is relevant when establishing healthcare priorities. Logistic regressions were used to explore the relation of respondents’ sociodemographic characteristics and health related behaviours in the likelihood of agreeing with the patients engaged in risky behaviour deserve a lower priority.

**Results:** Using illegal drugs was the behaviour most penalized (65.5%) followed by heavy drinkers (61.5%) and smoking (51.0%). The slight penalization was the unhealthy dieting (29.7%). The sociodemographic characteristics had different impact in penalization of the risks’ behaviours. Moreover, the respondents who support the idea that unhealthy lifestyles should have a lower priority, all strongly agreed that the smoking habit (OR=36.05; 95% CI: 8.72, 149.12), the unhealthy diets (OR=12.87; 95% CI: 3.21, 51.53), drink alcohol in excess (OR=20.51; 95% CI: 12.09, 85.46) and illegal drug use (OR=73.21; 95% CI: 9.78, 97.83) must have a lower priority in the access to healthcare.

**Conclusions:** The respondents accept the notion of rationing healthcare based on lifestyles.

## Introduction

A criterion that has gained importance during the last years supports the concept of equality of substantive opportunities, by taking into account the importance of personal responsibility when allocating the healthcare resources^1-3^. There is an increasing amount of information about the relation between health and lifestyles. According to WHO^4^, 68% of the 56 million deaths worldwide in 2012 were caused by non-communicable diseases and are projected to increase to 52 million in 2030. Four major non-communicable diseases (cardiovascular diseases, cancer, chronic respiratory diseases and diabetes) are responsible for 82% of the non-communicable disease deaths^4^. This has much to do with lifestyle habits, such as smoking, alcohol consumption and unhealthy dieting^5,6^.

Portuguese population has been performing poorly in terms of individual responsibility with their health. The national nutritional and physical activity survey, conducted in 2016, concluded that the Portuguese daily food intake was unbalanced, rich in sodium and sugar, poor in vegetables and fruits^7^. Furthermore, the Portuguese are mostly sedentary, with only 27% engaging in one-hour of moderate daily exercise, or half an hour of intensive exercise^7^.This combination may explain that more than half of the Portuguese population is obese or at risk of becoming obese. In relation to smoking habits, 20% of the Portuguese population (aged 15 yr or over) were regular smokers in 2014. The tobacco consumption was responsible for 19.9% of all of the respiratory disease deaths, 18.6% of the total cancer deaths and 15.2% of the total cardiovascular deaths^8^. Portugal had the highest alcohol consumption rates, at the European level, for almost all of the indicators investigated^9^. Finally, the prevalence of the illicit substance use throughout life, in 2012, was at 9.5% for the Portuguese population aged 15-64 years^9^.

This level of incidence of health damaging behaviours is particularly worrying as Portugal insofar as there is enormous pressure to reduce public expenditure on health. In this context we may raise a quite relevant question: ‘Due to the scarcity of the healthcare resources, will it be fair to ration healthcare resources according to each person’s degree of contributing to a particular illness?’

There is a growing empirical literature on the views of the general public regarding the equitable of treating patients differently based on their lifestyles^10-15^. The results seem to be inconsistent. While some studies suggest that the general public would indeed less prioritize those considered in some way responsible for their ill-health^10-12,16^, others do not find such evidence^13-15^. Besides, risk behaviours seem not to be equally penalized. Few studies have, however, directly and systematically explored simultaneously different health behaviours^17,18^.

This study examined whether different personal responsibilities could be taken into consideration when allocation healthcare resources in Portugal, characterized by a universal coverage healthcare system, and whether attitudes toward the penalization of risk behaviours vary among individuals with different sociodemographic characteristics and health related habits. We focused on four risk behaviours: smoking, consumption in excess of alcohol beverage, unhealthy dieting habits and illegal drug use.

## Methods

### Questionnaire

We developed an online survey and made it available on various social networks for six months, during 2015. We obtained 296 valid answers. The sample covered the population aged 18 yr and older living in Portugal. Before the implementation of the questionnaire we tested it through a previous sample (with different qualifications, professions and age), in order to verify whether the questions were clearly understood and analyse the overall degree of answers variability. The questionnaire was also validated by experts in the health economy and management. As will be described below (Table 1), our sample approaches the profile of the Portuguese population of most of the variables considered. We applied the test of the validity and reliability for the dependent variables of the questionnaire and we got a high internal reliability (Cronbach's alpha=0.841).

The questionnaire comprises four sections developed elsewhere^18^and adapted to Portuguese reality. Section A included the sociodemographic characteristics – gender, age, marital status, monthly income, degree and the employment status. Section B comprises health related habits: smoking status, alcohol consumption habits, consumption of fruits and vegetables, engagement in physical activities, fast food consumption, taking prescribed medication and acute illness. Section C included five general statements concerning the: level of satisfaction with life (one statement), self-reported health status (two statements), recognition that risk behaviours are harmful for health (one statement) and relationship between unhealthy lifestyles and lower priority in access to healthcare (one statement). In this section, respondents expressed the extent of their agreement on a five-point Likert scale (1-completely disagree, 2 - partly agree, 3 -neutral, 4 -partly disagree, 5 -completely disagree). In section D respondents were faced with the following question: “If rationing healthcare is necessary, do you think that the following risk behaviours should determine the priority loss?” The options “yes” and “no” were given. The risk behaviours were: smoking, overeating/unhealthy dieting, heavy drinking and illicit drug use. This last section constitutes the dependent variable while the other three sections reported the independent variables (Table A1).

Respondents were presented with an information sheet and gave their consent for the use of the data.

### Data analysis

All the analyses were performed with STATA (version 14). All statistical tests were two-sided and level of significance was set at 0.05. The logistic regressions were used to explore the determinants of the likelihood of agreeing (or disagreeing) with the notion that engaging in risk behaviours (smoking, alcohol abuse, unhealthy dieting and illegal drug use) should justify a lesser priority to healthcare. These models are more appropriate than the least squares model (OLS)^19^. Four models where conducted, with the dummy dependent variable assuming the value of 1, whenever respondents agreed that patients engaged in risk behaviour deserve a lesser priority.

## Results

### Sample

In the resulting sample of 296 respondents, 57.1% were female. The average age of the sample was 36 years; 58.1% were married and the majority were employed (90.5%). In terms of the monthly income, 18.9% received less or equal than 1000EUR, 34.8% between 1001 EUR and 2000 EUR, 24.0% between 2001EUR and 3000 EUR and 22.3% above 3000 EUR. Concerning qualifications, 1.4% of the respondents had a basic level, 12.8% had secondary education and 63.2% had higher education (Table 1).

**Table 1:** Descriptive statistics

**Independent variables**	**Number**	**Percent**
Sociodemographic variables		
Gender		
Female	169	57.1
Male	127	42.9
Age (yr)		
<25	38	12.8
25-35	112	37.8
36-45	95	32.1
46-55	39	13.2
56-68	12	4.1
Marital status		
Single	101	34.2
Married	172	58.1
Divorced	22	7.4
Widow	1	0.3
Monthly income (€)		
≤1000	56	18.9
1001-2000	103	34.8
2001-3000	71	24.0
>3000	66	22.3
Educational degree		
Primary education	4	1.4
Secondary education	38	12.8
Higher studies (Graduation)	187	63.2
Master degree or PhD	67	22.6
Employment situation		
Student/unemployed/housewife	43	14.5
Employed/self-employed	253	85.5
Smoking status		
No	214	72.3
Yes	64	21.6
Occasionally	18	6.1
Alcohol consumption		
No	117	39.5
Yes	32	10.8
Occasionally	147	49.7
Fruit or vegetables consumption		
Rarely or never	2	0.7
1-2 times a month	5	1.6
1-2 times a week	28	9.5
3-5 times a week	95	32.1
6-7 times a week	166	56.1
Engage in physical activities		
Rarely or never	73	24.7
1-2 times a month	56	18.9
1-2 times a week	98	33.1
3-5 times a week	62	20.9
6-7 times a week	7	2.4
Fast food consumption		
Rarely or never	116	39.2
1-2 times a month	149	50.3
1-2 times a week	27	9.1
3-5 times a week	2	0.7
6-7 times a week	2	0.7
Taking prescribed (or not) medication		
Rarely or never	130	43.9
1-2 times a month	93	31.4
1-2 times a week	17	5.7
3-5 times a week	14	4.7
6-7 times a week	42	14.3
Acute illness ^a^		
No	134	45.3
Yes	162	54.7
Satisfaction with life		
Strongly disagree	1	0.3
Disagree	21	7.1
Neither agree nor disagree	15	5.4
Agree	165	55.7
Strongly agree	93	31.5
A healthy person		
Strongly disagree	1	0.3
Disagree	13	4.4
Neither agree nor disagree	14	4.7
Agree	177	59.8
Strongly agree	91	30.8
Level of health can be affected by my behaviour		
Strongly disagree	0	0.0
Disagree	8	2.7
Neither agree nor disagree	5	1.7
Agree	86	29.1
Strongly agree	197	66.5
Unhealthy lifestyles should have a lower priority in treatments
Strongly disagree	65	22.0
Disagree	87	29.4
Neither agree nor disagree	59	19.9
Agree	48	16.2
Strongly agree	37	12.5
**Dependent variables**	**Number**	**Percent**
Smoking		
Yes	151	51.0
No	145	49.0
Unhealthy diet		
Yes	88	29.7
No	208	70.3
Excess alcohol		
Yes	182	61.5
No	114	38.5
Drug consumption		
Yes	194	65.5
No	102	34.5

^a^ Acute illness are those that have an accelerated course, ending with convalescence or death for example, cardiovascular diseases, cancer, among others

Although our sample may not be fully representative of the Portuguese society at large, it is similar in terms of the respondents’ main characteristics - gender, age, marital status and monthly income.

Descriptive statistics of the four dependent variables (smoking; overeating/unhealthy dieting, alcohol abuse and illicit drug use) can be found in the bottom panel of Table 1. Using illegal drugs was the behaviour most penalized (65.5%) followed by heavy drinkers (61.5%) and smoking (51.0%). In the opposite direction appears the slight penalization imposed on the unhealthy dieting (29.7%).

The results from the logistic regression models are summarized in Tables 2-5.

### Model 1 – Dependent Variable: Smoking

Results of Table 2 suggest that women penalized smoking habit, in relation to those who do not have this habit, less than men (odds ratio (OR)=-0.31; 95% confidence interval (95% CI): -0.15, -0.63). In turn, the respondents with high monthly income penalize more the smokers’ patients than the respondents with lower monthly income (OR=2.13; 95% CI: 0.69, 6.55).

**Table 2:** Association between agree that smoking habit should lost the priority in access to healthcare (To evaluate the possibility of correlation, we test relations between income and degree - Corr (Monthly Income, Education) = 0.0887.)

**Variables**	**Odds ratio (95% CI)**	***P *** **value**
Age (yr)	0.97 (0.93, 1.01)	0.231
Gender		
Female	1.00	
Male	-0.31 (-0.15, -0.63)	0.001
Marital status		
Single	1.00	
Married	2.70 (1.13, 6.44)	0.025
Divorced	1.10 (0.23, 5.17)	0.903
Widow	2.02 (0.43, 6.07)	0.813
Monthly income (€)		
≤1000	1.00	
1001-2000	0.95 (0.35, 2.54)	0.915
2001-3000	1.93 (0.63, 5.94)	0.251
>3000	2.13 (0.69, 6.55)	0.003
Educational degree		
Primary education	1.00	
Secondary education	4.01 (0.16, 97.95)	0.395
Higher studies (Graduation)	5.56 (0.24, 131. 38)	0.287
Master degree or PhD	4.37 (0.17, 109. 24)	0.370
Employment Situation		
Student/unemployed/housewife	1.00	
Employed or self-employed	0.40 (0.11, 1.44)	0.161
Smoker		
No	1.00	
Yes	-0.50 (-0.21, -1.17)	0.002
Occasionally	0.17 (0.04, 0.72)	0.216
Alcohol consumption		
No	1.00	
Yes	0.64 (0.18, 2.30)	0.004
Occasionally	1.94 (0.93, 4.05)	0.039
Fruits and/or vegetables consumption		
Rarely or never	1.00	
1-2 times a month	0.24 (0.02, 3.02)	0.272
1-2 times a week	0.60 (0.06, 6.10)	0.667
3-5 times a week	0.66 (0.07, 5.91)	0.706
6-7 times a week	0.56 (0.06. 6.81)	0.000
Engage in physical activities		
Rarely or never	1.00	
1-2 times a month	1.07 (0.41, 2.78)	0.886
1-2 times a week	0.76 (0.27, 2.12)	0.600
3-5 times a week	0.95 (0.07, 12.93)	0.969
6-7 times a week	0.63 (0.22, 1.10)	0.391
Fast food consumption		
Rarely or never	1.00	
1-2 times a month	0.35 (0.11, 1.14)	0.082
1-2 times a week	0.86 (0.37, 3.12)	0.610
3-5 times a week	0.75 (0.06, 8.93)	0.869
6-7 times a week	1.27 (0.62, 2.62)	0.518
Taking prescribed (or not) medication		
Rarely or never	1.00	
1-2 times a month	1.38 (0.32, 6.00)	0.671
1-2 times a week	0.78 (0.13, 4.64)	0.787
3-5 times a week	1.08 (0.35, 3.37)	0.892
6-7 times a week	1.21 (0.56, 2.61)	0.621
Acute illness ^a^		
No	1.00	
Yes	1.03 (0.53, 2.00)	0.931
Satisfaction with life		
Strongly disagree	1.00	
Disagree	0.22 (0.04, 1.09)	0.063
Neither agree nor disagree	1.56 (0.33, 7.32)	0.576
Agree	1.20 (0.56, 2.57)	0.643
Strongly agree	1.26 (0.60, 3.07)	0.313
A healthy person		
Strongly disagree	1.00	
Disagree	3.40 (0.40, 29.23)	0.265
Neither agree nor disagree	1.51 (0.31, 7.41)	0.610
Agree	2.04 (0.92, 4.53)	0.080
Strongly agree	1.03 (0.81, 3.43)	0.041
Level of health can be affected by my behaviour
Strongly disagree	1.00	
Disagree	4.40 (0.50, 19.23)	0.665
Neither agree nor disagree	2.42 (0.41, 6.41)	0.610
Agree	0.75 (0.08, 7.52)	0.810
Strongly agree	0.43 (0.05, 3.97)	0.021
Unhealthy lifestyles should have a lower priority in treatments
Strongly disagree	1.00	
Disagree	2.63 (1.00, 6.91)	0.650
Neither agree nor disagree	11.09 (3.49, 35.20)	0.320
Agree	19.07 (5.96, 61.03)	0.000
Strongly agree	36.05 (8.72, 149.12)	0.000
Constant	0.36 (0.00, 35.35)	0.662

^a^ Acute illness are those that have an accelerated course, ending with convalescence or death for example, cardiovascular diseases, cancer, among others

Respondents that smoke (OR=-0.50; 95% CI: -0.21, -1.17) in relation to non-smokers are less likely to agree with the smoking patients’ priority loss when accessing healthcare. On the opposite side, we have the respondents that drink (OR=0.64; 95% CI: 0.18, 2.30) and drink occasionally (OR=1.94; 95% CI: 0.93, 4.05) in relation to those who do not drink have more propensity to defend that the smokers should lose priority over non-smokers. Besides, the respondents that consume 6-7 times a week fruits and/or vegetables (OR=0.56; 95% CI: 0.06, 6.81) in relation to respondents that do not consume fruits and/or vegetables have more propensity to penalize the smoker patients. Furthermore, the respondents that strongly agree (OR=1.03; 95% CI: 0.81, 3.43) with the statement that “I consider myself a healthy person” and strongly agree (OR=36.05; 95% CI: 8.72, 149.12) with “Individuals with unhealthy lifestyles should have a lower priority in treatments” defend that the smoking habit warrants a lower priority in the access to healthcare.

### Model 2 – Dependent Variable: Unhealthy Diet

Elderly respondents (OR=-0.94; 95% CI: -0.89, -0.98) tend to agree less with the notion that patients with unhealthy food habits deserve loosing priority in accessing to healthcare (Table 3). On the other side, the married respondents (OR=3.61; 95% CI: 1.43, 9.11), in relation to singles, and those that have high monthly income (OR=1.49; 95% CI: 0.45, 4.91), in relation to respondents with low income, agree more with the notion that patients with unhealthy food habits (when compared with respondents with healthy diet) deserve losing priority in accessing to healthcare.

**Table 3:** Association between agree that unhealthy diet should lost the priority in access to healthcare (To evaluate the possibility of correlation, we test relations between income and degree - Corr (Monthly Income, Education) = 0.0887)

**Variables**	**Odds ratio (95% CI)**	***P *** **value**
Age (yr)	-0.94 (-0.89, -0.98)	0.004
Gender		
Female	1.00	
Male	1.33 (0.65, 2.71)	0.433
Marital status		
Single	1.00	
Married	3.61 (1.43, 9.11)	0.007
Divorced	1.60 (0.30, 8.50)	0.583
Widow	0.50 (0.23, 3.50)	0.674
Monthly income (€)		
≤1000	1.00	
1001-2000	0.53 (0.18, 1.53)	0.239
2001-3000	2.34 (0.73, 7.47)	0.150
>3000	1.49 (0.45, 4.91)	0.002
Educational degree		
Primary education	1.00	
Secondary education	5.12 (0.18, 141.33)	0.335
Higher studies (Graduation)	5.40 (0.21, 138.32)	0.308
Master degree or PhD	1.92 (0.07, 53.78)	0.702
Employment Situation		
Student/unemployed/housewife	1.00	
Employed or self-employed	0.57 (0.16, 1.97)	0.370
Smoker		
No	1.00	
Yes	1.06 (0.39, 2.89)	0.912
Occasionally	0.50 (0.11, 2.25)	0.364
Alcohol consumption		
No	1.00	
Yes	1.40 (0.34, 5.84)	0.645
Occasionally	1.10 (0.52, 2.34)	0.806
Fruits and/or vegetables consumption		
Rarely or never	1.00	
1-2 times a month	0.06 (0.01, 0.68)	0.123
1-2 times a week	0.06 (0.01, 0.60)	0.317
3-5 times a week	0.06 (0.01, 0.54)	0.012
6-7 times a week	0.42 (0.24, 1.34)	0.032
Engage in physical activities		
Rarely or never	1.00	
1-2 times a month	1.28 (0.46, 3.523	0.637
1-2 times a week	1.11 (0.36, 3.42)	0.857
3-5 times a week	2.07 (0.23, 18.90)	0.520
6-7 times a week	1.43 (0.45, 4.54)	0.541
Fast food consumption		
Rarely or never	1.00	
1-2 times a month	0.44 (0.11, 1.86)	0.266
1-2 times a week	1.32 (0.31, 2.77)	0.867
3-5 times a week	1.43 (0.66, 3.29)	0.996
6-7 times a week	1.49 (0.68, 3.27)	0.317
Taking prescribed (or not) medication		
Rarely or never	1.00	
1-2 times a month	2.72 (0.70, 10.59)	0.150
1-2 times a week	0.67 (0.11, 4.18)	0.667
3-5 times a week	3.27 (0.97, 10.98)	0.056
6-7 times a week	2.08 (0.88, 4.93)	0.095
Acute illness*		
No	1.00	
Yes	0.79 (0.40, 1.58)	0.509
Satisfaction with life		
Strongly disagree	1.00	
Disagree	1.16 (0.22, 6.13)	0.865
Neither agree nor disagree	0.84 (0.16, 4.52)	0.842
Agree	1.23 (0.55, 2.76)	0.612
Strongly agree		
A healthy person		
Strongly disagree	1.00	
Disagree	0.27 (0.02, 3.86)	0.334
Neither agree nor disagree	0.80 (0.16, 4.05)	0.784
Agree	1.18 (0.52, 2.67)	0.689
Strongly agree	0.47 (0.32, 4.86)	0.534
Level of health can be affected by my behaviour
Strongly disagree	1.00	
Disagree	1.92 (0.20, 10.64)	0.884
Neither agree nor disagree	1.15 (0.62, 3.67)	0.699
Agree	0.98 (0.10, 9.64)	0.984
Strongly agree	-0.36 (-0.04, -3.28)	0.203
Unhealthy lifestyles should have a lower priority in treatments
Strongly disagree	1.00	
Disagree	1.12 (0.35, 3.63)	0.849
Neither agree nor disagree	5.95 (1.72, 20.50)	0.205
Agree	8.75 (2.63, 29.09)	0.000
Strongly agree	12.87 (3.21, 51.53)	0.000
Constant	1.69 (0.02, 184.14)	0.826

^a^ Acute illness are those that have an accelerated course, ending with convalescence or death for example, cardiovascular diseases, cancer, among others

Within the scope of health related habits, the respondents with a healthier diet, that is to say, those who consume 3-5 times a week (OR=0.06; 95% CI: 0.01, 0.54) or consume 6-7 times a week (OR=0.42; 95% CI: 0.24, 1.34) fruits and vegetables penalize more the access to healthcare for the patients with unhealthy diet. Finally, the respondents who support, strongly agree (OR=12.87; 95% CI: 3.21, 51.53) in relation to strongly disagree the idea that unhealthy lifestyles should have a lower priority in treatments, defend that the patients with unhealthy diets must have a lower priority in the access to healthcare.

### Model 3 – Dependent Variable: Alcohol abuse

Elderly respondents (OR=-0.98; 95% CI: -0.93, -1.03) agree less with the penalization of patients drinkers in priority of treatment (Table 4). The respondents with a high monthly income (OR=5.97; 95% CI: 1.60, 22.24) in relation to those who have a lower income and the respondents employed or self-employed (OR=0.20; 95% CI: 0.05, 0.79) in relation to non-employed or unemployed students or homemakers defend more the penalization of heavy drinkers. We also observe that the respondents that smoke (OR=0.62; 95% CI: 0.26, 1.46) and smoke occasionally (OR=0.11; 95% CI: 0.02, 0.64) in relation to non-smokers, who consume alcohol (OR=0.33; 95% CI: 0.08, 1.27) in relation to respondents that do not consume alcohol, who consume 6-7 times a week fruits and/or vegetables (OR=10.05; 95% CI: 0.53, 16.06) in relation to respondents that do not consume fruits and/or vegetables, who have more physical activity (OR=0.21; 95% CI: 0.07, 0.64) in relation to those who practice less sports and who are on medication 1-2 times a month (OR=10.69; 95% CI: 1.40, 81.49) in relation to rarely or never, agreed more with the policy of granting a lower priority to healthcare access for the heavy drinkers. Moreover, the respondents that strongly agree (OR=20.51; 95% CI: 12.09, 85.46) with the notion of setting a lower priority to patients engaged in unhealthy lifestyles, defend more giving a lower priority to healthcare access for the heavy drinkers.

**Table 4:** Association between agree that drinking alcohol in excess should lost the priority in access to healthcare (To evaluate the possibility of correlation, we test relations between income and degree - Corr (Monthly Income, Education) = 0.0887)

**Variables**	**Odds ratio (95% CI)**	***P *** **value**
Age (yr)	-0.98 (-0.93, -1.03)	0.003
Gender		
Female	1.00	
Male	1.12 (0.52, 2.42)	0.769
Marital status	1.00	
Single	1.99 (0.79, 5.03)	0.144
Married	3.45 (0.73, 16.4)	0.120
Divorced	2.03 (0.04, 0.70)	0.820
Widow		
Monthly income (€)	1.00	
≤1000	3.59 (1.25, 10.27)	0.017
1001-2000	3.40 (1.00, 11.59)	0.050
2001-3000	5.97 (1.60, 22.24)	0.032
>3000		
Educational degree	1.00	
Primary education	8.79 (0.25, 313.15)	0.233
Secondary education	10.62 (0.31, 360.67)	0.189
Higher studies (Graduation)	6.98 (0.19, 254.74)	0.290
Master degree or PhD		
Employment Situation		
Student/unemployed/housewife	1.00	
Employed or self-employed	0.20 (0.05, 0.79)	0.022
Smoker		
No	1.00	
Yes	0.62 (0.26, 1.46)	0.002
Occasionally	0.11 (0.02, 0.64)	0.014
Alcohol consumption		
No	1.00	
Yes	0.33 (0.08, 1.27)	0.001
Occasionally	1.07 (0.50, 2.32)	0.858
Fruits and/or vegetables consumption
Rarely or never	1.00	
1-2 times a month	5.27 (0.29, 94.82)	0.259
1-2 times a week	11.75 (0.73, 18.00)	0.082
3-5 times a week	3.71 (0.29, 47.52)	0.313
6-7 times a week	10.05 (0.53, 16.06)	0.012
Engage in physical activities		
Rarely or never	1.00	
1-2 times a month	0.26 (0.09, 0.76)	0.014
1-2 times a week	0.37 (0.12, 1.15)	0.085
3-5 times a week	0.14 (0.01, 2.00)	0.149
6-7 times a week	0.21 (0.07, 0.64)	0.003
Fast food consumption		
Rarely or never	1.00	
1-2 times a month	1.33 (0.94, 4.08)	0.260
1-2 times a week	1.22 (0.98, 3.46)	0.553
3-5 times a week	0.23 (0.06, 0.87)	0.130
6-7 times a week	0.88 (0.40, 1.95)	0.756
Taking prescribed (or not) medication
Rarely or never	1.00	
1-2 times a month	10.69 (1.40, 81.49)	0.022
1-2 times a week	0.59 (0.09, 3.89)	0.583
3-5 times a week	1.07 (0.31, 3.61)	0.919
6-7 times a week	1.58 (0.69, 3.61)	0.281
Acute illness*		
No	1.00	
Yes	1.50 (0.72, 3.11)	0.282
Satisfaction with life		
Strongly disagree	1.00	
Disagree	0.20 (0.04, 0.94)	0.042
Neither agree nor disagree	2.16 (0.37, 12.72)	0.396
Agree	1.36 (0.58, 3.17)	0.482
Strongly agree	4.16 (3.06, 13.44)	0.878
A healthy person		
Strongly disagree	1.00	
Disagree	3.88 (0.38, 39.61)	0.252
Neither agree nor disagree	2.28 (0.40, 13.09)	0.356
Agree	1.51 (0.65, 3.53)	0.338
Strongly agree	3.25 (2.43, 8.64)	0.446
Level of health can be affected by my behaviour
Strongly disagree	1.00	
Disagree	0.32 (0.27, 6.5)	0.738
Neither agree nor disagree	1.11 (0.22, 16.22)	0.532
Agree	0.77 (0.07, 8.5)	0.829
Strongly agree	1.08 (0.11, 10.72)	0.947
Unhealthy lifestyles should have a lower priority in treatments
Strongly disagree	1.00	
Disagree	2.92 (1.17, 7.31)	0.222
Neither agree nor disagree	23.38 (6.86, 79.62)	0.340
Agree	47.74 (12.15, 187.54)	0.000
Strongly agree	20.51 (12.09, 85.46)	0.000
Constant	0.02 (0.00, 3.13)	0.126

^a^ Acute illness are those that have an accelerated course, ending with convalescence or death for example, cardiovascular diseases, cancer, among others

### Model 4 – Dependent Variable: Illegal Drug use

Elderly respondents (OR=0.96; 95% CI: 0.91, 1.00) and those with a higher monthly income (OR=2.88; 95% CI: 0.85, 9.80) in relation to those with less income, defend the idea of penalizing illicit drug users in treatment priority (Table 5). On the opposite side are the respondents that smoke (OR=-0.37; 95% CI: -0.16, 0.84) in relation to non-smokers and those who consume alcohol (OR=-0.62; 95% CI: -0.18, -2.17) in relation to the respondents that do not consume alcohol agree with the idea of penalizing illicit drug users in treatment priority. The respondents that consume 6-7 times a week fruits and/or vegetables (OR=12.44; 95% CI: 0.62, 132.26) in relation to respondents that do not consume agree to penalize the priority to the patients that have a drug use habit (in relation to the patients that do not have this habit). Lastly, the respondents who strongly agree (OR=73.21; 95% CI: 9.78, 97.83) with the idea that unhealthy lifestyles should have a lower priority in treatments, defend that the drug use habit warrants a lower priority in the access to healthcare.

**Table 5:** Association between agree that illegal drug user should lost the priority in access to healthcare

**Variables**	**Odds ratio (95% CI)**	***P *** **value**
Age	0.96 (0.91, 1.00)	0.044
Gender		
Female		
Male	-0.59 (-0.28, -1.25)	0.167
Marital status		
Single	1.00	
Married	1.99 (0.82, 4.82)	0.129
Divorced	1.03 (0.23, 4.54)	0.968
Widow	1.33 (0.53, 4.34)	0.457
Monthly income (€)		
≤1000	1.00	
1001-2000	1.52 (0.57, 4.04)	0.402
2001-3000	1.61 (0.50, 5.12)	0.422
>3000	2.88 (0.85, 9.80)	0.011
Educational degree		
Primary education	1.00	
Secondary education	11.46 (0.41, 317.09)	0.150
Higher studies (Graduation)	15.39 (0.57, 411.92)	0.103
Master degree or PhD	13.29 (0.47, 378.91)	0.130
Employment Situation		
Student/unemployed/housewife	1.00	
Employed or self-employed	0.20 (0.05, 0.81)	0.025
Smoker		
No	1.00	
Yes	-0.37 (-0.16, -0.84)	0.018
Occasionally	0.31 (0.07, 1.47)	0.141
Alcohol consumption		
No	1.00	
Yes	-0.62 (-0.18, -2.17)	0.014
Occasionally	1.61 (0.75, 3.45)	0.218
Fruits and/or vegetables consumption		
Rarely or never	1.00	
1-2 times a month	16.28 (1.05, 253.26)	0.046
1-2 times a week	13.44 (0.98, 184.78)	0.052
3-5 times a week	6.21 (0.55, 70.29)	0.140
6-7 times a week	12.44 (0.62, 132.26)	0.012
Engage in physical activities		
Rarely or never	1.00	
1-2 times a month	0.69 (0.26, 1.83)	0.450
1-2 times a week	0.58 (0.20, 1.72)	0.329
3-5 times a week	0.30 (0.02, 4.04)	0.361
6-7 times a week	0.56 (0.20, 1.61)	0.284
Fast food consumption		
Rarely or never	1.00	
1-2 times a month	0.35 (0.11, 1.13)	0.080
1-2 times a week	0.42 (0.22, 1.82)	0.165
3-5 times a week	0.29 (0.03, 1.86)	0.349
6-7 times a week	0.83 (0.39, 1.78)	0.632
Taking prescribed (or not) medication		
Rarely or never	1.00	
1-2 times a month	1.36 (0.25, 7.41)	0.724
1-2 times a week	0.65 (0.09, 4.62)	0.669
3-5 times a week	1.52 (0.45, 5.10)	0.497
6-7 times a week	1.07 (0.48, 2.39)	0.862
Acute illness ^a^		
No	1.00	
Yes	1.44 (0.71, 2.92)	0.316
Satisfaction with life		
Strongly disagree	1.00	
Disagree	0.28 (0.06, 1.26)	0.097
Neither agree nor disagree	1.75 (0.26, 11.91)	0.568
Agree	0.87 (0.39, 1.98)	0.747
Strongly agree	0.56 (0.43, 2.89)	0.659
A healthy person		
Strongly disagree	1.00	
Disagree	4.47 (0.66, 30.39)	0.126
Neither agree nor disagree	1.25 (0.25, 6.18)	0.784
Agree	2.04 (0.90, 4.62)	0.089
Strongly agree	3.22 (0.74, 20.22)	0.421
Level of health can be affected by my behaviour
Strongly disagree	1.00	
Disagree	1.82 (0.46, 2.32)	0.179
Neither agree nor disagree	1.32 (0.22, 13.19)	0.440
Agree	2.18 (0.23, 20.30)	0.494
Strongly agree	2.58 (0.31, 21.81)	0.384
Unhealthy lifestyles should have a lower priority in treatments
Strongly disagree	1.00	
Disagree	1.64 (0.71, 3.79)	0.245
Neither agree nor disagree	18.30 (5.36, 62.50)	0.260
Agree	27.16 (7.09, 103.97)	0.000
Strongly agree	73.21 (9.78, 97.83)	0.000
Constant	0.02 (0.00, 3.05)	0.131

^a^ Acute illness are those that have an accelerated course, ending with convalescence or death for example, cardiovascular diseases, cancer, among others. To evaluate the possibility of correlation, we test relations between income and degree - Corr (Monthly Income, Education) = 0.0887.

## Discussion

In Portugal, as in most western societies, the rapidly rising healthcare expenditures, coupled with the increasing pressure to maintain a balanced state budget may jeopardize the universal healthcare access. One possible way of differentiating the patients’ healthcare access is through taking into account their specific lifestyle behaviours. This selection maybe particularly relevant in Portugal insofar as Portuguese citizens seem to fail in meeting their societal obligation as is contemplated in the Constitution of Portuguese Republic: “Everyone has the right to health protection and the duty to protect and promote it”^20^.

In this context, we conducted an exploratory study to identify whether Portuguese respondents accept lifestyles (smoking, unhealthy dieting, alcohol abuse and illicit drug use) as a criterion to access to healthcare and, if so, to identify which is the most condemned one. One key strength of our study is that it is the first time that views about establishing priorities between patients supported by lifestyles choices in Portuguese society have been assessed in a systematic way. The results seem to be, in general, quite robust. The notion that individuals with unhealthy lifestyles should indeed have lower priority in treatments was statistically significant for all of the detrimental behavioural patterns. This result is consistent with the desert-based distribution principle of healthcare.

One common desert issue in healthcare context is the lifestyle choices of people whose ill-health is related to those choices.^3^This idea rests in the principle of equality of substantive opportunity for health,^1^according to which inequality in achieving health outcomes may be acceptable if it arises from unhealthy lifestyle choices for which individuals ought to be held responsible. An implication is that patients who are deemed partly responsible for their own illness should receive lower priority for treatment. The results indicate a wider consensus, among respondents, in giving lower priorities to patients that use illegal drugs, that drink alcohol in excess and that smoke. From all the lifestyles, illicit drug use was the most penalized. Heavy drinking was more penalized than smoking, which is consistent with some previous research results^14^. Following an unhealthy diet, by contrast, was the lifestyle less penalized. We can only speculate with regard to the reasons of such a lower penalty rate. One possible explanation is that it is commonly known that maintaining a healthy diet is quite expensive preventing numerous people from following it. So, consuming a cheaper diet, rich in fat and sugar may be excused. This notion seems to be corroborated by the results in Table 3. Those respondents who believed that harmful behaviours affect health were also those that most disagreed with limiting the access to healthcare to patients not following a healthy diet. The same notion was revealed by the respondents following a healthy diet. Recent data suggests that during 2015-2016, 10.1% of Portuguese families experienced food insecurity. This means that they had difficulty in providing sufficient amounts of food for the entire family, due to the lack of financial resources^7^.

Furthermore, respondents’ attitudes with regard to the penalization of risk behaviours seem to adhere to the rational choice theory. With the exception of the unhealthy diet habit, respondents seem to prefer the distribution mechanism that is most advantageous for them. This was notorious with patients’ smoking and drinking habits. Smoking respondents (heavy drinkers) disagreed that smoking (alcohol abuse) should bar the access to healthcare. These results converge to international findings.^15^ Our analysis also shows that older respondents are significantly less likely to penalize unhealthy diet practices and heavy alcohol beverage consumption. Both risky behaviours are more prevalent among the elderly. Recent data revealed that the prevalence of obesity in Portugal is much higher among the elderly (39.2%, compared with the national average of 22.3%) and the consumption of alcohol beverages is particularly higher for the same group (298 g/d, compared with the national average of 146 g/d).^7^Contrary to previous findings, there is no evidence of gross-behaviour effects.^18^ Our results seem to suggest that smokers or heavy drinkers are more willing to agree that heavy drinkers or smokers should lose priority in access to healthcare. On the other hand, respondents with higher levels of income are significantly more likely to agree that risky behaviour should influence the establishing of priorities to healthcare access, penalize all the harmful behavioural patterns. This may be explained by the fact that they contribute more to the health system, through taxes, and as such think the money should be most effectively spent.

The results should be interpreted with appropriate caution, however, given the non-random nature of the sample. The findings cannot be generalized to the Portuguese people at large. According to recent data, in Portugal, there is a predominance of female inhabitants (52.6%), the average age of the population is 41.8 yr, 40.5% are married and the monthly income is 1083 EUR (Pordata, 2017). The sample was better educated than the general population (only 17.1% of the population has higher education)^21^. Furthermore, the data were collected through an online questionnaire. We are quite aware that the mode of administrating the questionnaire has important repercussions for the subsequent respondents’ sample. The online surveys method enables a large number of responses to be collected quickly and relatively cheaply, but raises concerns with regard to the quality of data obtained and denies the researcher a representative population sample. Even so, in the recent years there has been an increasing interest in collecting data online^22-24^. There is a lack of literature examining the impact of this mode of administrating a survey, in order to elicit societal preferences. The majority of the studies find an overall, broadly similar response throughout all the different survey administration modes^22-24^. Besides this sampling limitation, there are also concerns with regard to submitting the questions related to setting healthcare priorities to individuals under the universal coverage health system.

## Conclusions

Overall, Portuguese respondents accept the notion of rationing healthcare based on lifestyles. This serves to explain the public’s acceptance of the measures undertaken by the Portuguese government during the last few years in order to control tobacco and alcohol abuse (through indirect tax increases and the prohibition of indoor smoking) and, more recently, by controlling the excessive intake of sugars in non-alcohol beverages. Our analysis suggests that future policies, advocating rationing based on individual responsibility, might be supported by the Portuguese population. We believe that given the high incidence of risk behaviours in Portuguese society it would be worth investing in health literacy policies. This policy could decrease the incidence of chronic diseases by informing citizens about the social costs of their lifestyles and the potential hazard on their health status.

In follow-up research, it would be useful to explore the views of health professionals about using lifestyles as a criterion to establish priorities between patients. Moreover, it would be useful to extend this study to a representative sample of the Portuguese population and then compare the opinions of general population with those of health professionals. It would also be useful to conduct international comparative research using a common study design – either this one or another common format – in order to explore existing cultural differences and trace the patterns of common distributive principles.

## Acknowledgements

We are deeply grateful to Duje Petricevic for useful comments and suggestions.

## Conflict of interest statement

The authors declare that they have no conflict of interest.

## Funding

The authors disclosed receipt of any financial support for the research, authorship, and/or publication of this article.

## Highlights

- More than half of the Portuguese respondents penalized unhealthy lifestyles (smoking, consumption of alcoholic beverages, unhealthy diet and illegal drug use).
- Illegal drug users were the most penalized group, followed by those consuming alcohol excessively, as well as those smoking. However, unhealthy dieting was only residually penalized.
- Sociodemographic characteristics and health related behavior play a different role when penalizing the unhealthy lifestyles.
